# Selective Vagus Nerve Stimulation as a Therapeutic Approach for the Treatment of ARDS: A Rationale for Neuro-Immunomodulation in COVID-19 Disease

**DOI:** 10.3389/fnins.2021.667036

**Published:** 2021-04-13

**Authors:** Svetlana Mastitskaya, Nicole Thompson, David Holder

**Affiliations:** Department of Medical Physics and Biomedical Engineering, University College London, London, United Kingdom

**Keywords:** vagus nerve, neuromodulation, inflammation, cytokine storm, ARDS, COVID**-19**

## Abstract

Acute respiratory distress syndrome (ARDS) is the most severe form of acute lung injury. It is induced by sepsis, aspiration, and pneumonia, including that caused by SARS coronavirus and human influenza viruses. The main pathophysiological mechanism of ARDS is a systemic inflammatory response. Vagus nerve stimulation (VNS) can limit cytokine production in the spleen and thereby dampen any systemic inflammation and inflammation-induced tissue damage in the lungs and other organs. However, the effects of increased parasympathetic outflow to the lungs when non-selective VNS is applied may result in bronchoconstriction, increased mucus secretion and enhance local pulmonary inflammatory activity; this may outweigh the beneficial systemic anti-inflammatory action of VNS. Organ/function-specific therapy can be achieved by imaging of localized fascicle activity within the vagus nerve and selective stimulation of identified organ-specific fascicles. This may be able to provide selective neuromodulation of different pathways within the vagus nerve and offer a novel means to improve outcome in ARDS. This has motivated this review in which we discuss the mechanisms of anti-inflammatory effects of VNS, progress in selective VNS techniques, and a possible application for ARDS.

## Introduction

Acute respiratory distress syndrome (ARDS) is a fulminant condition which may result in a mortality rate of more than 40% (Diamond et al., 2020). It may be caused by direct lung injury due to bacterial or viral pneumonia, inhalation of smoke, toxic chemicals, or aspiration of gastric contents, or by indirect injury due to septic shock, acute pancreatitis, burn injury, or major trauma (Wong et al., 2019). Whether induced by pulmonary or extra-pulmonary insult, ARDS is caused by pulmonary injury which manifests as interstitial and alveolar edema, severe hypoxemia, endothelial injury, and an acute systemic inflammatory response which may rapidly progress to respiratory and multi-system failure (Matthay et al., 2019; Diamond et al., 2020). ARDS secondary to virally driven pneumonia is the predominant cause of mortality from SARS-CoV-2 infection (Mehta et al., 2020; Zhang et al., 2020).

### Systemic Inflammatory Response in Severe COVID Patients

In COVID-19 disease, angiotensin-converting enzyme 2 (ACE2) on the surface of the cells serves as an entry point for SARS-CoV-2 virus (Hoffmann et al., 2020). It is richly expressed in lung epithelial cells, as well as in the heart, gastrointestinal tract (GIT) and kidneys (Samavati and Uhal, 2020). Elevated plasma levels of Angiotensin II (as a result of ACE2 internalization upon viral entry) in critically ill COVID-19 patients (Ni et al., 2020) may stimulate monocyte recruitment from the spleen. The monocytes migrate to the infected tissues within 24 h where they contribute to the initial inflammatory damage (Swirski et al., 2009) and promote neutrophilic activation and migration into the interstitial and alveolar spaces. If the innate immune system fails to clear the pathogen or repair the lungs from the insult, the overactivation of the systemic immune response results in release of the pro-inflammatory cytokines interleukin-1α (IL-1α), IL-6, IL-1β, tumor necrosis factor alpha (TNF-α), and interferon gamma (IFN-γ). This is commonly termed a “cytokine storm” (Mehta et al., 2020). Analysis of the lung immune microenvironment using bronchoalveolar lavage fluid from severe and moderate COVID-19 patients showed that highly inflammatory monocyte-derived splenic macrophages prevail in the excessive inflammatory response in the lungs from patients with ARDS (Liao et al., 2020). These macrophages of splenic origin are active producers of chemokines and cytokines which promote neutrophilic migration into alveolar space and hyperactivation. The activated neutrophils release proteases and reactive oxygen species which contribute to endo- and epithelial integrity disruption, the further increase of vascular permeability with protein-rich exudate floating in the alveoli, and formation of hyaline membranes (Matthay et al., 2019). Homeostatic mechanisms opposing the effects of systemic inflammation include endogenous glucocorticoid secretion and the release of anti-inflammatory cytokines such as IL-10 (Johnston and Webster, 2009); however, they may be insufficient to limit this fulminant inflammatory cascade.

### Anti-inflammatory Therapy of Cytokine Storm and ARDS in COVID-Disease

Anti-inflammatory medications aiming at reducing the cytokine storm and systemic inflammation in COVID-19 patients include non-steroidal anti-inflammatory drugs, glucocorticoids, immunosuppressants, and antagonists of inflammatory cytokines (such as IL-6R antibodies, TNF inhibitors, IL-1R antagonists, etc.). Dexamethasone was shown to be effective in improving survival in critical and severe cases of COVID-19 infection—including those requiring mechanical ventilation due to ARDS (Horby et al., 2021). Until the COVID-19 pandemic, there was no conclusive evidence for the advantage of the steroids use for the prevention or treatment of ARDS associated with other causes, and it still needs to be established whether the benefits of prolonged low dose corticosteroids outweigh the short and long-term risks, including delayed recovery (Mokra et al., 2019). Another promising therapy using Tocilizumab, a monoclonal antibody against the receptor of pro-inflammatory cytokine IL-6, emerged as an alternative treatment for COVID-19 patients with a risk of acute systemic inflammatory response and in need of mechanical ventilation (Guaraldi et al., 2020). However, anti-inflammatory medications, such as corticosteroids, may delay the elimination of the virus and increase the risk of secondary infections in immunocompromised patients (Zhang et al., 2020). Drugs targeting a particular cytokine can only inhibit a specific inflammatory factor, and thus may not be effective enough in limiting the effects of other cytokines of significance. Therefore, choosing the correct time window for anti-inflammatory therapy and identifying the patients that are most likely to benefit from immunosuppression remains a critical issue. Patients with severe COVID-19 disease could be screened for hyperinflammation using laboratory trends (e.g., increased ferritin, decreased platelet counts, or erythrocyte sedimentation rate) to identify a subgroup of patients for whom immunosuppression could improve survival (Mehta et al., 2020). It is evident, however, that identification of such patients and initiation of an anti-inflammatory therapy is required well before their condition progresses to severe stages, as ARDS is an advanced manifestation of a cytokine storm, which by that point may already have caused irreversible damage.

## Vagus Nerve Stimulation

**FIGURE 1:**
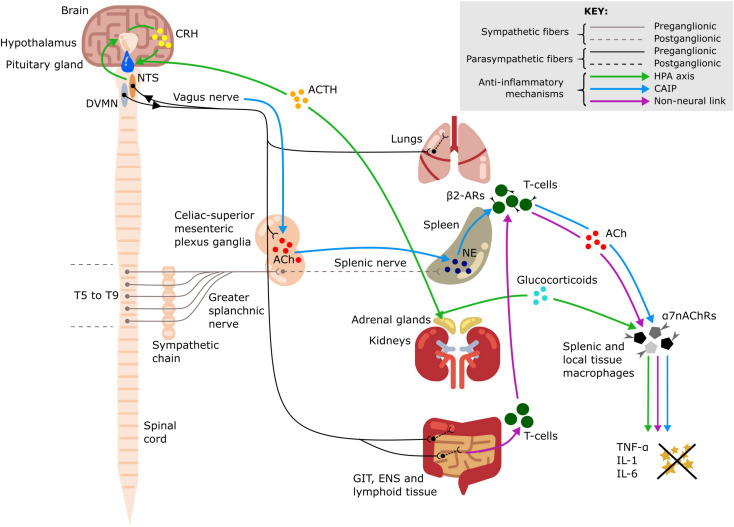
Anti-inflammatory pathways of the vagus nerve. A schematic representation of the anti-inflammatory pathways of the vagus nerve including the hypothalamic-pituitary-adrenal (HPA) axis (green arrows), the cholinergic anti-inflammatory pathway (CAIP) (blue arrows) and the non-neural link between the vagus nerve and spleen (purple arrows). All three pathways result in attenuation of pro-inflammatory cytokine production, including tumor necrosis factor-α (TNF-α), interleukin-1 (IL-1), and interleukin-6 (IL-6). Dorsal motor nucleus of the vagus nerve (DVMN), nucleus of the solitary tract (NTS), gastrointestinal tract (GIT), enteric nervous system (ENS), thoracic vertebrae (T5–T9), acetylcholine (ACh), NE (norepinephrine), corticotropin-releasing hormone (CRH), adrenocorticotrophic hormone (ACTH), β2-adrenergic receptors (β2-ARs), and α7–nicotinic ACh receptors (α7nAChRs).

The vagus nerve is the main component of the parasympathetic nervous system. It innervates the majority of visceral organs, including the pharynx, larynx, tracheobronchial tree and lungs, heart, esophagus, stomach, liver, gallbladder, pancreas, small intestine, and proximal colon (Thompson et al., 2019). Importantly, the vagus nerve plays an integral role in the connection between the nervous and immune systems (Figure 1; Borovikova et al., 2000; Kressel et al., 2020). Therefore, it is of particular interest in neuromodulation of inflammation. Vagus nerve stimulation (VNS) has indirect inhibitory effects on the cytokine production in the spleen even though there is no evidence for direct cholinergic (vagal) innervation of the spleen in humans (Verlinden et al., 2019). The existing methods of cervical VNS in human patients employ electrical stimulation of the entire nerve with circumferential wire loops. The applied electrical current activates the entire vagus and all its fibers, both afferent and efferent, which results in preferential activation of sensory (afferent) fibers because they have lower activation threshold. This can cause multiple unwanted side effects, such as nausea, cough, and headache, which may limit the VNS tolerability and efficiency (Howland, 2014).

**FIGURE 2:**
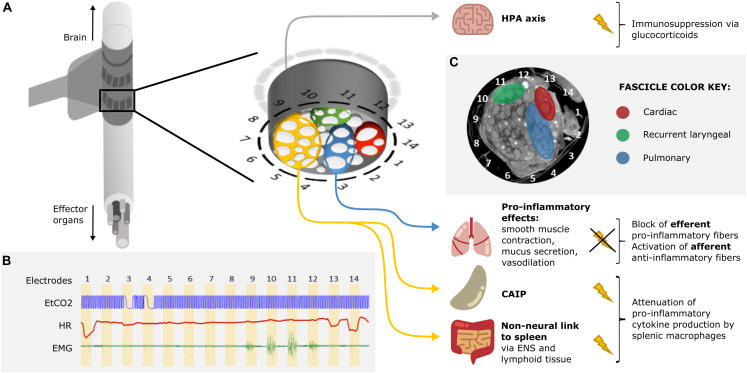
Proposed approach for VNS in ARDS treatment. **(A)** A schematic of a silicone rubber cuff with 14 circumferential electrodes wrapped around the vagus nerve. Inset: a more detailed schematic of the cross-section of the vagus nerve surrounded by electrodes. The fascicles (white) of the nerve (gray) are grouped into four regions identified by selective stimulation and by subsequent micro-computed tomography (microCT): recurrent laryngeal (green), cardiac (red), pulmonary (blue), and the rest of the nerve with fascicles suspected to innervate the abdominal viscera (orange). Stimulation of the orange region of the nerve would result in the activation of the cholinergic anti-inflammatory pathway (CAIP) and the non-neural link between the vagus and the spleen, via the enteric nervous system (ENS) and lymphoid tissue of the gastrointestinal tract (GIT), via vagal efferents resulting in the attenuation of pro-inflammatory cytokine production. Stimulation of the vagal afferents would activate the hypothalamic-pituitary-adrenal (HPA) axis which would result in immunosuppression via glucocorticoids. Selective blocking of the pulmonary fascicles would prevent activation of pulmonary efferent fibers and the inflammation-potentiating effects of smooth muscle contraction, increased mucus secretion and vasodilation in the lungs. Overall, suppression of the immune reaction would be achieved. **(B)** Identification of organ-specific fascicles with a quick round of selective VNS. The pulmonary, cardiac, and recurrent laryngeal fascicles are localized by sequential stimulation of the radial sections of the nerve via electrode pairs 1–14 (stimulation for 30 s on each pair followed by 30 s recovery period) and reading out of physiological parameters: changes in respiration (end-tidal CO2, EtCO2), heart rate (HR), and contraction of the neck muscles (electromyography, EMG), accordingly. Pulmonary fascicles are located next to electrode pairs 3 and 4 (bradypnea); cardiac fascicles next to pairs 1, 13, and 14 (bradycardia); and recurrent laryngeal fascicles next to pairs 10 and 11 (maximal EMG signal). **(C)** A microCT cross-section of a vagus nerve at the cervical level with identified regions containing recurrent laryngeal (green), cardiac (red), and pulmonary (blue) fascicles (unpublished data, study on the left vagus nerve in pigs).

An attractive possibility is to undertake selective stimulation of the cervical vagus nerve. Unfortunately, until recently, this was limited as the functional anatomy of fascicles in the vagus nerve was almost entirely unknown. In our group at University College London, we have developed a method to image localized fascicle compound action potential activity with Electrical Impedance Tomography (EIT) using a silicone rubber cuff with 14 circumferential electrodes (Figure 2A; Ravagli et al., 2019, 2020). Identified fascicles can then be selectively stimulated using two such electrode rings spaced 3 mm apart (Figure 2B; Aristovich et al., 2021). Our studies suggest the organotopic organization of the fascicles of the cervical vagus nerve in large mammals (sheep and pigs). Until now, three regions – namely cardiac, pulmonary and recurrent laryngeal—were localized within the cervical region of the vagus nerve and can be selectively modulated (Figures 2B,C). Work is in progress to achieve the same imaging and selective modulation of the other organs supplied by the vagus nerve. The findings are being independently validated by micro-computed tomography (microCT) tracing of fascicles from their end-organs (Thompson et al., 2020).

### Systemic Anti-inflammatory Effects of VNS

Potent systemic anti-inflammatory effects of VNS suggest that VNS could be a promising alternative immunomodulatory treatment for patients with ARDS (Van Westerloo et al., 2006; Krzyzaniak et al., 2011; Supplementary Table 1). VNS was shown to attenuate the release of pro-inflammatory cytokines, modulate coagulation, prevent circulatory failure, and thus decrease organ dysfunction and improve survival in animal models of sepsis and endotoxemia (Borovikova et al., 2000; Van Westerloo et al., 2006). Clinical studies also demonstrated immunomodulatory effects of VNS—suppression of inflammation and improvement of clinical symptoms in rheumatoid arthritis (Koopman et al., 2016), intractable epilepsy (Majoie et al., 2011), atrial fibrillation (Stavrakis et al., 2015), and Crohn’s Disease (Bonaz et al., 2016). These effects are mediated by the following mechanisms (Figure 1):

#### Hypothalamic-Pituitary-Adrenal Axis

Vagus nerve afferents express IL-1β receptors at the level of paraganglia and can therefore sense local and systemic inflammation (Bonaz et al., 2016). Activation of these afferents leads to glutamate release in the nucleus of the solitary tract (NTS). The NTS sends adrenergic projections to the paraventricular nucleus of the hypothalamus, which contains a population of corticotropin-releasing hormone (CRH) neurons (Hosoi et al., 2000). CRH then acts on the anterior pituitary gland and stimulates the release of adrenocorticotropic hormone into systemic circulation (Hosoi et al., 2000) with an ultimate effect on the adrenal cortex and increased secretion of glucocorticoids which are very effective in suppressing the immune system (Fleshner et al., 1995; Bonaz et al., 2016).

#### Cholinergic Anti-inflammatory Pathway (CAIP)

This is a potent anti-inflammatory pathway in the spleen which is indirectly activated by stimulation of vagus nerve efferent fibers. The efferent innervation of the spleen comprises noradrenergic sympathetic fibers within the splenic nerve (Verlinden et al., 2019). Some vagal preganglionic neurons terminate in the celiac-superior mesenteric ganglia, where much of the postganglionic sympathetic nerve supply to the spleen is derived (Kressel et al., 2020). The axons of vagal preganglionic neurons form varicose-like structures surrounding individual splenic nerve cell bodies and thereby modulate the activity of the splenic nerve (Kressel et al., 2020). Acetylcholine (ACh) released from vagus nerve efferents in the celiac ganglion activates postsynaptic α7-nicotinic ACh receptors (α7nAChRs) of the splenic nerve (Vida et al., 2011). This results in the release of norepinephrine in the spleen where it acts on β2-adrenergic receptors of splenic CD4^+^T-cells that also release ACh. T-cell derived ACh acts on α7nAChRs of splenic macrophages which leads to a decrease of pro-inflammatory cytokine production via inhibition of the transcription factor NF-kB p65 (Rosas-Ballina et al., 2008). The spleen is the major source of cytokine production in conditions of systemic inflammation such as sepsis; thus, the cholinergic anti-inflammatory pathway (CAIP) is a potent mechanism exploited by VNS for treatment of inflammatory diseases. Direct stimulation of the efferent vagus nerve inhibits the synthesis of pro-inflammatory cytokines in liver, spleen, and GIT, and also decreases their levels in systemic inflammatory responses to endotoxemia, ischemia, sepsis and other diseases (Rosas-Ballina et al., 2008; Dos Santos et al., 2011). It has been shown that the pro-inflammatory cytokine production is attenuated by VNS, but the release of IL-10, which has counter-inflammatory actions, is unaffected (Borovikova et al., 2000).

#### Non-neural Link From Vagus to Spleen

A critical review of the CAIP pathway is provided in the work by Martelli et al. (2014) who also suggest a non-neural mechanism linking the activity of the vagus nerve to the decreased production of pro-inflammatory cytokines by splenic macrophages via activation of α7nAChRs receptors. The vagus nerve provides extensive innervation of secondary lymphoid tissue in the GIT and increased parasympathetic stimulation of these lymphoid depots mobilizes their ACh-synthesizing T-cells. The circulating T-cells are sequestered by the spleen, where they release ACh acting on α7nAChRs expressed by splenic macrophages (Martelli et al., 2014).

#### Inhibition of Tissue Macrophage Activity

A significant additional contribution to the anti-inflammatory effects of VNS is mediated by vagal efferent fibers which synapse on intrinsic neurons of the enteric nervous system in the GIT (Matteoli et al., 2014) and terminate in other visceral organs, including the liver (Borovikova et al., 2000) and lungs. Tissue macrophages contribute to the production of the pro-inflammatory cytokines released during a systemic inflammatory response; during an excessive response, this contributes to the cytokine storm and results in damage to multiple organs (Johnston and Webster, 2009). ACh released by vagal efferents acts on α7nAChRs of local tissue macrophages in the gut which leads to decreased production of the main pro-inflammatory cytokine, TNF-α (Matteoli et al., 2014). In the same way, the resident immune cells of the lungs—including alveolar macrophages, epithelial cells and activated infiltrating neutrophils—can be affected by ACh acting on their α7nAChRs to slow down the local inflammatory reaction and alleviate lung injury (Su et al., 2010).

### Pulmonary Effects of VNS

Non-selective VNS will stimulate parasympathetic fibers to the lungs but this inadvertent activation may not be beneficial. It will activate pulmonary cholinergic efferents which have pro-inflammatory effects. Parasympathetic efferent stimulation leads to activation of muscarinic ACh (mACh) receptors on airway smooth muscle, glands, and vasculature which results in airway smooth muscle contraction, increased mucus secretion and vasodilation (Gosens et al., 2006). Whereas mucus secretion is an important mechanism of innate defense in airways, its excessive production and accumulation in alveoli during the inflammatory process impairs the blood-gas barrier, potentiates hypoxia and inflammatory injury (Fahy and Dickey, 2010). The predominant immune cells present in the air space are alveolar macrophages. ACh was found to stimulate these cells which resulted in the release of chemotactic activity for inflammatory cells, such as neutrophils, monocytes, and eosinophils (Sato et al., 1998). By blocking mACh receptors in mice, the production of cytokines contributing to inflammatory infiltrate and tissue damage in the lungs was inhibited (Gori et al., 2019).

On the other hand, stimulation of pulmonary afferent A-fibers (pulmonary stretch receptors) causes dyspnea and reflexly decreased parasympathetic tone, resulting in effects opposite to stimulation of pulmonary efferents—bronchodilation and decreased mucus secretion (Kubin et al., 2006). It is unclear if stimulation of the pulmonary vagal fascicles will preponderantly affect afferent or efferent fibers in the lungs. Selective VNS would be necessary to tease out whether pulmonary fibers should be stimulated or blocked to ameliorate the cytokine storm and improve outcome by modifying other parasympathetic controlled variables in ARDS.

### VNS in Experimental Models of ARDS

In a rat model of venom-induced ARDS, vagal efferent stimulation was protective against Mesobuthus tamulus (MBT), but not against oleic acid (OA)-induced ARDS (Akella and Deshpande, 2015). The protective effect was explained by increased surfactant secretion and activation of the anti-inflammatory pathway. Interestingly, VNS was only effective in the MBT model—this model is characterized not only by pulmonary injury, but also by systemic cardiovascular alterations. Perhaps, the beneficial role of VNS, which was evident from prolonged survival of animals in this model, is attributed to cardiovascular effects of increased parasympathetic tone rather than its anti-inflammatory action on the lungs.

Beneficial effects of vagal efferent stimulation were reported in ventilator-induced ARDS (Brégeon et al., 2011; Dos Santos et al., 2011) and in peritonitis-induced lung injury (Boland et al., 2011), but not in other models of ARDS (sepsis and ventilation; Kox et al., 2012). In a rat model of endotoxemia-induced pulmonary inflammation potentiated by mechanical over-ventilation (Kox et al., 2012), no benefit of VNS was observed, which questions the clinical applicability of stimulation of the CAIP in systemically inflamed patients admitted to the ICU where mechanical ventilation is initiated. In this study, the vagus nerve was not transected; therefore, both afferent and efferent fibers were stimulated, and the stimulation was applied to the entirety of the nerve, with the potential detrimental effects of pulmonary efferent fiber stimulation outweighing the anti-inflammatory action of VNS. Additionally, the timing of VNS could be very critical—in this study, VNS was applied when septic shock was fully developed. It may be that the magnitude of the systemic reaction was already too high to be affected by VNS.

### Proposed Approach for VNS in ARDS Treatment

It is evident that VNS assists in improving outcomes and mortality of immune dysregulation through its anti-inflammatory action (Dos Santos et al., 2011; Bonaz et al., 2016; Koopman et al., 2016; Liu et al., 2017). Existing techniques stimulate the entire nerve and often result in unwanted side effects or lack therapeutic effect due to insufficient intensity. We hypothesize that it may be possible to improve outcome in ARDS by selective VNS. This could permit employment of more optimal stimulation paradigms as they need not be limited by off-target side effects, and it may be that differential modulation of pulmonary vagal tone may yield additional benefits. Various techniques of selective VNS have been suggested, including anodal block (Tosato et al., 2007), depolarizing pre-pulses (Vuckovic et al., 2008), kilohertz electrical stimulation block (Patel et al., 2017), fiber-selective stimulation (McAllen et al., 2018) and spatially selective stimulation (Aristovich et al., 2021). Anodal block, depolarizing pre-pulses and fiber-specific stimulation allow for efficient mitigation of laryngeal side effects (Vuckovic et al., 2008) but not enough selectivity with regards to target organs or effectors. Unlike fiber-specific stimulation, spatially selective VNS accounts for the organotopic arrangement of fibers within the cervical vagus nerve (Figure 2). It provides more precise targeting than fiber-specific VNS and has been demonstrated to mitigate side effects and successfully elicit organ-specific responses (Ordelman et al., 2013; Plachta et al., 2014; Aristovich et al., 2021).

### Invasive vs Non-invasive VNS

Non-invasive VNS does not require surgical intervention, and thus improves the safety and tolerability of VNS. Currently, there are two types of non-invasive VNS—transcutaneous and auricular VNS. In transcutaneous VNS (tVNS), the stimulating electrodes are applied to the skin surface over the sternocleidomastoid muscle in the neck (Yap et al., 2020). Auricular VNS (aVNS) targets the sensory auricular branch of the vagus nerve in the ear. This method makes use of the auricular-vagal reflex which involves the auricular concha, vagus nerve, NTS and the dorsal motor nucleus of the vagus nerve (Yap et al., 2020). Both tVNS and aVNS have been shown to elicit similar therapeutic effects as VNS (Hein et al., 2013; Koopman et al., 2016; Subramanian et al., 2020; Yap et al., 2020).

However, both tVNS and aVNS do not allow for the modulation of the activity of the vagus nerve organ- or function-specifically—their disadvantage compared to invasive VNS. Invasive VNS uses a surgically implantable device wrapped around the cervical vagus nerve which allows for the suggested selective VNS with a specifically designed electrode cuff. Selective activation of certain fibers of the vagus nerve, such as certain desired pathways or functions (e.g., CAIP), but not others (e.g., pulmonary fibers), is unlikely to be feasible with tVNS or aVNS which only allow for indiscriminate stimulation of all fibers (Yuan and Silberstein, 2016).

### Possible Risks and Challenges

Vagus nerve stimulation is known to have a number of off-target effects (voice alteration, cough, dyspnea, dysphagia, etc.) which are mostly stimulation-related (Howland, 2014). With the proposed use of selective stimulation, these adverse effects could be avoided. The risk of bronchoconstriction and increased mucus secretion associated with stimulation of pulmonary efferent fibers would need to be monitored and avoided as to not contribute to ARDS pathogenesis further. In addition, laryngeal and esophageal muscle contractions in intubated patients would need to be prevented (Zalvan et al., 2003), as it would be a risk for mechanical damage to the nerve and upper airway obstruction. Implanting a VNS device in critically ill patients in ICU can be challenging. Ideally, the decision on the VNS device implantation should be informed by early laboratory signs of systemic inflammation, and the patients who are likely to progress to ARDS would undergo the VNS device implantation prior to the full development of cytokine storm and ARDS.

## Conclusion

The ability of VNS to contain immune activation at the crucial stages of a nascent response whilst not impairing the specific immunity against infectious agents is highly advantageous in treating ARDS and other immune dysregulation diseases. In contrast to immunosuppressive therapy, activation of the CAIP via abdominal efferents of the vagus nerve synapsing in the celiac-superior mesenteric ganglionic complex is desirable to attenuate the over-production of pro-inflammatory cytokines—the pathophysiological feature in ARDS. However, the effect of stimulation on the pulmonary fibers needs to be considered as it is likely that this will potentiate inflammation by activation of bronchoconstriction and mucus secretion, negating the beneficial anti-inflammatory effects of CAIP activation. Selective modulation of the vagus nerve could offer the greatest chance of improving ARDS outcomes by employing independent activation or block of the splenic and pulmonary immune pathways as needed.

## Author Contributions

SM and DH conceptualized and supervised the work. SM and NT wrote the manuscript. NT designed the figures. All authors contributed to the article and approved the submitted version.

## Conflict of Interest

The authors declare that the research was conducted in the absence of any commercial or financial relationships that could be construed as a potential conflict of interest.
