# Supplementary material for: Selective Vagus Nerve Stimulation as a Therapeutic Approach for the Treatment of ARDS: A Rationale for Neuro-Immunomodulation in COVID-19 Disease
Source: Front Neurosci. 2021 Apr 13;15:667036. doi: 10.3389/fnins.2021.667036 (PMC8076564; doi:10.3389/fnins.2021.667036)
Supplement: Supplementary file 1 [file Data_Sheet_1.PDF]

Supplementary Table 1: Parameters of VNS and major outcomes of treatment in clinical studies.

Condition/Study	*Type of VNS device*	*Current*	*Duty cycle*	*Outcome*
**Heart (atrial fibrillation)**				
TREAT-AF Paroxysmal AF (Stavrakis et al., 2020)	Auricular VNS: ear clip attached to the tragus	20 Hz, 1 mA below the discomfort threshold	4.2%: 1 h daily over 6 months	6 months: the median AF burden was 85% lower in the active arm (n=26) compared with the control arm (n=27), increase in HRV; TNF-α decreased by 23%.
**Crohn disease**				
NCT01569503 (Bonaz et al., 2016)	Left VN: helical cuff Cyberonics electrode (model 302)	1.25 mA, 500 µs pulse, 10 Hz	9%: 30 s on, 300 s off	5 out of 9 patients showed full clinical-biological-endoscopic remission; increased HRV; improvement in C-reactive protein values.
NCT02311660 (Bonaz et al., 2016)	Left VN: helical cuff Cyberonics electrode (model 302)	2 mA, 250 µs pulse, 10 Hz	9%: 30 s on, 300 s off	At 16 weeks, improvements in endoscopic scores and HRV in 6 out of 8 patients.
**Rheumatoid arthritis**				
NCT01552941 (Koopman et al., 2016)	Left VN: patients with VNS for epilepsy	1-2 mA, 500 µs pulse, 20 Hz	0.07-0.28%: 60 s 1-4 times daily	In 17 RA patients, decreased TNF-α, IL-1β, and IL-6 production and significantly improved clinical signs and symptoms for 84 days.
**Asthma**				
VNS for acute exacerbation of asthma (Miner et al., 2012)	Percutaneous stimulation of right VN	Median peak stim at 4.4 V (1-11.6 V range), 200 µs pulse, 25 Hz	One-off stim for 60 min	In 25 patients, rapid and progressive improvement of forced expiratory volume and perceived dyspnea.
**Experimental ARDS**				
Venom-induced lung injury: MBT and OA models (Akella and Deshpande, 2015)	Bilateral stimulation of transected vagi: efferent fibers only	10 V, 1 ms pulse, 5 Hz	5 min stim before venom administration, and repeated for 5 min 10 min after injection	Extended survival of MBT model but no effect on OA model.
Rats, ventilator-induced lung injury (Brégeon et al., 2011)	Bilateral efferent VNS (transected vagi): bipolar platinum electrodes	5–10 V, 2 ms pulse, 5–10 Hz	50%: 10 min on, 10 min off	Decreased inflammation in alveolar tissue (no lymphocyte infiltration), IL-6 concentration decreased both locally (lung extracts) and systemically (plasma). This was attenuated by alpha7 nicotinic ACh receptor antagonists.
Rats and mice, ventilator- and hemorrhagic shock induced lung injury (Dos Santos et al., 2011)	Bilateral stimulation of distal end of transected vagi Bipolar hook electrodes	1 V, 2 ms pulse, 5 Hz	20 min total	IL-6 values improved.

Two-hit model in rats: sepsis and mechanical damage to the lungs (ventilation at 15 cmH2O) (Kox et al., 2012)	Vagi intact, bilateral stimulation of both afferent and efferent fibers.	5 V, 2 ms pulse, 5 Hz	3 min total	No effect on pulmonary inflammation.
Acid-induced acute lung injury in rabbits (Liu et al., 2017)	Right vagus nerve (intact – both afferents and efferents)	5 V, 2 ms pulse, 1 Hz	15 min total after acid aspiration	Decreased levels of plasma IL-10 and pulmonary leucocyte infiltration; improved pulmonary function.

Supplementary References:

Akella, A, and Deshpande, SB (2015). Vagal efferent stimulation protects against Mesobuthus tamulus venom-induced acute respiratory distress syndrome in rats. *Toxicon* 108**,** 189-201. doi: 10.1016/j.toxicon.2015.10.013

Bonaz, B, Sinniger, V, Hoffmann, D, Clarençon, D, Mathieu, N, Dantzer, C, et al. (2016). Chronic vagus nerve stimulation in Crohn's disease: a 6-month follow-up pilot study. *Neurogastroenterology & Motility* 28**,** 948-953. doi: 10.1111/nmo.12792

Brégeon, F, Xeridat, F, Andreotti, N, Lepidi, H, Delpierre, S, Roch, A, et al. (2011). Activation of Nicotinic Cholinergic Receptors Prevents Ventilator-Induced Lung Injury in Rats. *PLOS ONE* 6**,** e22386. doi: 10.1371/journal.pone.0022386

Dos Santos, CC, Shan, Y, Akram, A, Slutsky, AS, and Haitsma, JJ (2011). Neuroimmune Regulation of Ventilator-induced Lung Injury. *American Journal of Respiratory and Critical Care Medicine* 183**,** 471-482. doi: 10.1164/rccm.201002-0314OC

Koopman, FA, Chavan, SS, Miljko, S, Grazio, S, Sokolovic, S, Schuurman, PR, et al. (2016). Vagus nerve stimulation inhibits cytokine production and attenuates disease severity in rheumatoid arthritis. *Proceedings of the National Academy of Sciences* 113**,** 8284. doi: 10.1073/pnas.1605635113

Kox, M, Vaneker, M, Van Der Hoeven, JG, Scheffer, G-J, Hoedemaekers, CW, and Pickkers, P (2012). Effects of Vagus Nerve Stimulation and Vagotomy on Systemic and Pulmonary Inflammation in a Two-Hit Model in Rats. *PLOS ONE* 7**,** e34431. doi: 10.1371/journal.pone.0034431

Liu, Y, Tao, T, Li, W, and Bo, Y (2017). Regulating autonomic nervous system homeostasis improves pulmonary function in rabbits with acute lung injury. *BMC pulmonary medicine* 17**,** 98-98. doi: 10.1186/s12890-017-0436-0

Miner, JR, Lewis, LM, Mosnaim, GS, Varon, J, Theodoro, D, and Hoffmann, TJ (2012). Feasibility of Percutaneous Vagus Nerve Stimulation for the Treatment of Acute Asthma Exacerbations. *Academic Emergency Medicine* 19**,** 421-429. doi: 10.1111/j.1553-2712.2012.01329.x

Stavrakis, S, Stoner, JA, Humphrey, MB, Morris, L, Filiberti, A, Reynolds, JC, et al. (2020). TREAT AF (Transcutaneous Electrical Vagus Nerve Stimulation to Suppress Atrial Fibrillation): A Randomized Clinical Trial. *JACC Clin Electrophysiol* 6**,** 282-291. doi: 10.1016/j.jacep.2019.11.008
